# Molecular epidemiology and pathogenicity of Wesselsbron virus circulating in Africa

**DOI:** 10.1016/j.virusres.2024.199499

**Published:** 2024-11-17

**Authors:** Martin Faye, Nicholas Di Paola, Moussa Dia, Amadou Alpha Sall, Ousmane Faye

**Affiliations:** a Virology Department, Institut Pasteur de Dakar, 36, Avenue Pasteur, 220 Dakar, Senegal; b Center for Genome Sciences, United States Army Medical Research Institute of Infectious Diseases, Fort Detrick, Frederick, Maryland 21702, USA

**Keywords:** Wesselsbron virus, Molecular epidemiology, Pathogenicity, Tropism West Africa

## Abstract

Wesselsbron is a neglected, mosquito-borne zoonotic disease transmitted by several species of virus-infected *Aedes* mosquitoes endemic to tropical regions in Africa. It affects primarily domestic livestock species with teratogenic effects, but can jump to humans. Herein, we investigated the molecular epidemiology of Wesselsbron virus in Africa using whole genome sequencing and structural analysis, and assessed its pathogenicity and tropism through *in vivo* experiments. A total of twenty-five isolates collected from three countries were successfully characterized. Our study is noteworthy by identifying, for the first time, inter-clade recombination events on the genome of Wesselsbron virus. However, more investigations on the precise molecular mechanisms conducting the occurrence of recombination on the genome of Wesselsbron virus, are warranted. The identification of polymorphisms on motifs of virulence and selection pressures on major proteins showed evidence of genetic evolution for Wesselsbron virus. The clade 1 was more pathogenic and neurotropic in suckling mice and the intramuscular route was found to be the best transmission mode. Our findings also provide new insights in the pathogenicity and tropism of Wesselsbron virus, which could be useful for prevention, preparedness and future outbreak response. Considering its high prevalence in mosquito populations and the increasing number of sporadic human cases, Wesselsbron virus merits more attention in terms of prevention and preparedness, as its mosquito vectors continue to globally expand and there is no vaccine.

## Highlights

• Twenty-five Wesselsbron virus isolates from Africa were successfully characterized.
• First identification of recombination events on the genome of Wesselsbron virus.
• The clade 1 Wesselsbron virus was more pathogenic and neurotropic in suckling mice.
• The intramuscular route was the best transmission mode for Wesselsbron virus.
• Our data provide new insights in the pathogenicity and tropism of Wesselsbron virus.

## 1 Introduction

First isolated in 1955 in South Africa, Wesselsbron virus (WSLV) is a neglected arbovirus widely distributed in Africa (Karabatsos, 1985). Belonging to the *Flaviviridae* family, this zoonotic mosquito-borne orthoflavivirus is responsible of a neglected disease of public health and economic importance, associated to teratogenic effects in lambs, abortion and mortality in pregnant ewes as the Rift valley fever (RVF) (Karabatsos, 1985; Weiss et al., 1956; Coetzer and Barnard, 1977). It is especially pathogenic to young animals and can cause high mortality (up to approximately 25%) among newborn lambs and kids (Weiss et al., 1956), resulting in severe economic losses and socio-economic impact on production, trade and food security. Although no major outbreak has been reported in humans yet, a total number of 31 sporadic human cases has been reported (Karabatsos, 1985; Diagne et al., 2017; Weyer et al., 2013).

As is characteristic of orthoflaviviruses, WSLV has a linear single-stranded, positive-sense RNA genome (Diagne et al., 2017). The WSLV genome is 10,681 nucleotides in length, encoding a single polyprotein (3406 amino acids) from which 11 viral proteins are generated (3 structural (Capsid (C), Envelop (E) and Membrane (prM/M)) and 7 non-structural proteins (NS1, NS2A, NS2B, NS3, NS4A, 2K, NS4B and NS5)) and flanked by 5′ and 3′ untranslated regions (UTRs) of 85 and 379 nt, respectively (Diagne et al., 2017). Despite its low genetic diversity (< 1%), two phylogenetic clades (Karabatsos, 1985 and Weiss et al., 1956) have been previously described in Africa (Diagne et al., 2017), with no spatial or geographical correlation (Weyer et al., 2013).

Although WSLV causes only a mild fever in adult goats, cattle and pigs (Mushi et al., 1998), it has also been associated with neurological damages in horses and was isolated from rodent's brain tissues (Diagne et al., 2017, Venter et al., 2010; Weyer et al., 2013). Infection in humans typically presents as an influenza-like illness including a short period of fever and a mild acute phase with rash, arthralgia and myalgia (Diagne et al., 2017; Swanepoel, 1989). However, occupational exposure in a laboratory worker has exhibited a more severe clinical hallmark including neurological complications such as intense headache, memory loss and severe muscle spasms, abdominal pain and liver tenderness (Weinbren, 1959).

Several isolations of WSLV have been reported from mosquito populations (Jupp, 1996; Kokernot et al., 1960; Kokernot et al., 1958; Diallo et al., 2005), livestock (Weiss et al., 1956; Durand et al., 2001), wildlife (McIntosh, 1980) and humans (Diagne et al., 2017; Weyer et al., 2013) and serological evidence of WSLV circulation in various hosts (Baba et al., 1995; Barnard, 1997; Kokernot et al., 1965; Smithburn et al., 1959a) has been previously described in Sub-Saharan countries and Thailand (Mushi et al., 1998; Gould et al., 2003). In addition, increases in geographical distribution of the *Aedes* vectors during the past two decades (Kraemer et al., 2015; Kayiwa et al., 2022), could have enhanced human exposure and potential risks of WSLV spillover in humans in tropical and subtropical areas in Africa, where knowledge and resources are limited. Although previous *in vivo* studies exhibited the virulence of WSLV in mice (Way et al., 1976) and its the neuro-invasion in ovine foetus (Oymans et al., 2020), more experimental investigations are warranted for better characterization of the viral pathogenicity and identification of the virus tropism.

To bring attention to a neglected mosquito-borne virus infecting humans, firstly, we assessed nucleotides and amino acid distances, informative sites on highly conserved motives of virulence found in related viruses, recombination events, selective pressures on West African WSLV sequences. In addition, we provided new insights in the molecular epidemiological, pathogenicity and tropism of WSLV circulating in Africa using *in vivo* experimental studies in mice, useful for the development of prevention countermeasures or treatment to this zoonotic virus.

## 2 Materials and methods

### 2.1 Ethical statement

Mosquito pools used in this study were collected in the frame of the national integrated surveillance program for Arbovirus in Senegal while archived clinical specimens were provided by the WHO Collaborating Centre for Arboviruses and Hemorrhagic Fevers in Institute Pasteur in Dakar, accredited for routine diagnostic, surveillance and animal research, according to IACUC animal guidelines (University of Iowa Principles for the Care and Use of Laboratory Animals (IACUC), 2013). The Senegalese national ethical committee approved the protocol as a less than minimal risk research, and written consent forms were not required. All viral isolations and experiments in mice were performed in accordance with the ARRIVE guidelines regarding the sample size of animals allocated to each group and the experimental procedures (Percie du Sert et al., 2020).

### 2.2 Virus stocks preparation

A total of twenty-eight WSLV stocks previously identified by inoculating *Aedes albopictus* continuous cell lines (C6-36) for 4 days, followed by a specific immunofluorescence assay (IFA) as previously described (Digoutte et al., 1992), were obtained from the collection of the WHO collaborating centre for arboviruses and viral haemorrhagic fevers (CRORA) at the Institut Pasteur de Dakar (IPD) in Senegal. Filtrated virus stocks were passed one time by intracerebral inoculation of newborn Swiss suckling mice (1-2 days old) in animal facilities at the IPD. Infected mice were followed daily for any signs of encephalitis (e.g., incoordination, ataxia, limb weakness/paralysis) and euthanized. Brain tissues from febrile suckling mice were collected, homogenized in L-15 medium (Gibco BRL, GrandIsland, NY), filtered and frozen at -80°C until use (Supplementary Table S1).

### 2.3 RNA extraction and real-time RT-PCR

Extraction of viral RNA from 140 microliter (µL) of virus stocks was performed with the QIAamp viral RNA mini kit (Qiagen, Heiden, Germany) according to manufacturer's instructions. Viral RNA was eluted in a final volume of 60 μL and frozen at -80°C prior to downstream applications. The RNA extracts were tested by real-time reverse-transcriptase quantitative polymerase chain reaction (RT-qPCR) using the qScript One-step RT-qPCR Kit (Quanta Biosciences, Gaithersburg, MD, USA) with specific WSLV primers and probe in a final volume of 25 μL following the previously established protocol (Faye et al., 2022).

### 2.4 Complete genome sequencing

Briefly, a depletion of the ribosomal RNA of the host was made followed by the synthesis of the complementary DNA and the random amplification of the DNA fragments by the SISPA method (Sequence-independent, single-primer amplification). Then the libraries were prepared with the Nextera XT Library Prep kit, according to the manufacturer's recommendations. The libraries were tagged using indexes (Nextera XT index kit V2, Illumina) and pooled at the same concentration. Whole-genome sequencing was performed with paired-end reads using the Illumina MiSeq reagent kit v2 (300 cycles) on an Illumina MiSeq instrument. To generate the consensus genomes, we used the fully open-source EDGE Bioinformatics software (Li et al., 2017).

### 2.5 Sequences analysis

Multiple alignments of WSLV nucleotide sequences available online including isolates from South-Africa and Senegal (Diagne et al., 2017) (www.ncbi.nlm.nih.gov/genbank/*),* were carried out by using Muscle algorithm (http://www.drive5.com/muscle/) (Edgar, 2004) within Unipro UGENE software (http://ugene.net/download.html) (Okonechnikov et al., 2012). Pairwise amino acid distances at genes level were analyzed among of the new characterized Senegalese sequences and between them and the previously available complete genomes from Senegal (SN) (GenBank accession: KY056256-8) and South Africa (ZA) (GenBank accession: EU707555, JN226796 and MK163943). In addition, amino acid substitutions were also assessed on the highly conserved motifs of virulence previously described on major proteins of the genome of mosquito-borne flaviviruses (MBFVs) such as the E, NS1, NS3 and NS5 (Huhtamo et al., 2009; Scaramozzino et al., 2001; Kuno et al., 1998). Comparatively, conservation of these motifs was also assessed in insect-specific flaviviruses; (ISFs) (Culex flavivirus (CxFV) and Aedes flavivirus (AeFV)); (ISFs)) and in vertebrate-specific flaviviruses, also known as no known vector flaviviruses (NKVFs) (Modoc virus (ModV) and Rio Bravo virus (RBV)). Considering its close genetic relation with Yellow Fever virus (YFV) with respect to the E protein (Heinz and Stiasny, 2017) and the pathogenetic disease's similarity with Zika virus (ZIKV) regarding the teratogenic effect, the presence of motifs of virulence previously reported for YFV and ZIKV (Huang et al., 2014; Shan et al., 2020), was assessed in the newly characterized WSLV sequences.

### 2.6 Recombination and positive selection detection

The presence of recombination events was assessed in our dataset of complete genome sequences from Western and Southern Africa using seven methods (RDP, GENECONV, BootScan, MaxChi, Chimaera, SiScan, and 3Seq) implemented in the Recombination Detection Program (RDP4.97) (http://web.cbio.uct.ac.za/~darren/rdp.html) (Martin et al., 2015) and the Genetic Algorithms for Recombination detection (GARD) method implemented in Datamonkey web server (http://datamonkey.org) (Pond et al., 2005). Only breakpoints detected by at least five methods with a Bonferroni-corrected p-value <0.05 were reported. In addition, episodes of positive diversifying selection at gene level were analyzed using four different methods (SLAC, MEME, FUBAR, aBSREL, BUSTED) implemented in the HyPhy package from Datamonkey web server (Pond et al., 2005). An episode of positive selection was considered if it was detected by at least three different methods.

### 2.7 Molecular evolution and phylodynamics

Our dataset included sequences from Western and Southern Africa. Alignments were done in Geneious, version 11.1.4, using MAFFT, version 7.388. The Maximum-likelihood (ML) phylogeny was generated using PhyML version 3.3 with the GTR+ Γ model with 1000 bootstrap replicates. The ML phylogenetic tree was used for estimates of root-to-tip distances, regression slopes, and correlations using TempEst with the best-fitting root option (Rambaut et al., 2016). Tip divergence data were exported and mapped with linear regression (95% prediction interval) in Rstudio, version 3.2.3, with the R *Stats* and *ggplot2* packages (Wickham, 2016).

### 2.8 Viral fitness

#### 2.8.1 Virus stocks titration

Based on the recombination and phylogenetic data, two WSLV isolates including one from Senegal (ArD85094, NS1-NS2A recombinant and clade 1) and one from Mauritania (ArD140187, E-NS1 recombinant and clade 2) were selected for the in vivo experiments. The virus stocks were tested by plaque assay on green monkey kidney fibroblast (Vero) cells and characterized using Next-generation sequencing as described above. Information regarding the 2 isolates are gathered in Table 1.

**Table 1:** Description of isolates of Wesselsbron virus used in the experimental study

Isolate	Origin	Year of isolation	Specie	Virus stock titer (PFU/mL)	Clade	Accession number
ArD85094	Senegal	1987	*Aedes dalieli*	1.075×10^8^	1	PP445085
ArD140187	Mauritania	1995	*Aedes vexans*	2.000×10^8^	2	PP445099

#### 2.8.2 Mice inoculation

The experiments were performed in duplicates. Furthermore, 20 µL of viral doses containing 10^5^ PFU were diluted in 1X phosphate buffered saline (PBS) solution and all mice in the inoculated groups received the same dose. Two groups of twelve Swiss suckling mice (1-2 days old) and 2 groups of six 3-weeks old (wko) Swiss adult mice were inoculated by intraperitoneal (i.p) route while 2 groups of twelve Swiss suckling mice (1-2 days old) and 2 groups of six 3-wko Swiss adult mice were inoculated by intramuscular (i.m) route. Control groups including 3 Swiss suckling mice or 3-wko Swiss adult mice, received 20 µl of 1X PBS. The inoculated mice were monitored twice and daily until clinical signs of morbidity were observed, at which point monitoring was increased to three times daily over 21 days. Any 3-wko Swiss adult mouse that showed signs of morbidity at monitoring time points was euthanized with deep inhalational isoflurane followed by cervical dislocation according to IACUC animal guidelines (University of Iowa Principles for the Care and Use of Laboratory Animals (IACUC), 2013) and succumbed mice found during monitoring were also collected for further investigations. Brain punctures of succumbed suckling mice were also collected. If no sign of morbidity was observed at the monitoring points, one 3-wko Swiss adult mouse was euthanized in each inoculated group, at dpi 1, 7, 14 and 21 as previously described (University of Iowa Principles for the Care and Use of Laboratory Animals (IACUC), 2013). Mice surviving at dpi 21 were also euthanized as previously described (University of Iowa Principles for the Care and Use of Laboratory Animals (IACUC), 2013). Specimens were collected and stored at -80 ºC for further assessments. Comparisons of survival rates between inoculated and control groups were carried out using the log-rank (Mantel-Cox test) and Gehan-Breslow-Wilcoxon tests.

#### 2.8.3 Wesselsbron virus tropism

During euthanasia, mice were exposed to deep inhalational isoflurane and bled by cardiac puncture before cervical dislocation (Gould et al., 2003). In addition, brain, lungs, liver and kidneys’ tissues from euthanized mice or deceased mice at monitoring time points were collected for assessment of WSLV tropism. Organs tissues were homogenized in 1X PBS solution using the TissueLyser homogenizer (Qiagen, Germany). After centrifugation, the filtrated mixture supernatant was tested by real-time reverse-transcriptase quantitative polymerase chain reaction (RT-qPCR). The corresponding copy numbers were calculated using the equation obtained from a linear-regression analysis of a standard RNA as previously described (Faye et al., 2022). Differences in organ tissue infection between WSLV isolates were analysed using the one-way ANOVA test with a Geisser Green-house's epsilon correction in Prism7 (https://www.graphpad.com/scientific-software/prism/).

## 3 Results

### 3.1 Genetic distances

Out of the 28 isolates, 25 complete genome sequences from Senegal, Mauritania and Côte d'Ivoire, were generated (89.28%) with 10,808 nucleotides of length. The newly characterized sequences were submitted to Genbank (www.ncbi.nlm.nih.gov/genbank/) under accession numbers PP445078-PP445102 (Supplementary Table S1). To assess the genetic divergence of WSLV, pairwise genetic distances of coding sequences were evaluated at nucleotide and amino acid levels using a dataset of 31 WSLV isolates from Africa, including the 25 newly characterized sequences. Despite nucleotide distances ranging from 0 to 7%, the analyzed sequences showed genetic distances between 0 and 1% at amino acid level (data not shown), suggesting a low genetic diversity of WSLV sequences analyzed in this study.

### 3.2 Genetic motifs and informative sites on WSLV genome

Here, we described the location of main conserved amino acid motifs on major proteins (E, NS1, NS3 and NS5) of 31 WSLV isolates from Africa, including the 25 newly characterized sequences. Most of highly conserved amino acid motifs localized across E, NS1, NS3 and NS5 proteins of MBVFs were identified in the WSLV genomes, sometimes with presence of conservative amino acid mutations (positions highlighted in Black) or non-conservative amino acid mutations (positions highlighted in red) However, two non-conservative amino acid motifs were identified in the genome of all WSLV isolates, between positions 556–562 in the E protein (GHV**T**C**KA**) and between positions 692–699 in the NS1 (**EHS**WDFGS). Interestingly, although conserved in most of the WSLV isolates, an amino acid motif in the NS3 protein (SIAARGY) exhibited three non-conservative mutations in 1 isolate from Senegal and another from Côte d'Ivoire (**IM**A**DI**G**W)**. In addition, an amino acid motif in the NS5 protein (SRNSTHEMY) showed one non-conservative mutation in an isolate from Senegal (SRNSTHE**R**Y). The *in silico* analysis also showed that these MBFVs' amino acid motifs were mostly conserved in WSLV than in NKVFs and ISFs (Supplementary Table S2). Interestingly, the RGD (Arg-Gly-Asp) motif located in the FG loop of the MBVFs domain III of envelope protein (EDIII) (Huang et al., 2014), was identified as MGD (Met-Gly-Asp) between amino acid positions 357-359 of the E protein for all WSLV sequences (data not shown). In addition, the envelope protein V473M substitution (E-V473M; motif LL**M**WLGLN between aa positions 471-478) previously identified in ZIKV and associated with an increased neurovirulence, maternal-to-fetal transmission, and viremia to facilitate urban transmission (Martin et al., 2015), was identified at amino acid position 459 in the E protein of WSLV and replaced by the Methionine was replaced by an isoleucine (E-M459I; motif LL**I**WLGLN between aa positions 457-464) (Supplementary Figure S1).

### 3.3 Recombination

The assessment of recombination events was performed using the RDP4.97 program on a dataset of 31 WSLV isolates from Africa, including the 25 newly characterized WSLV genomes. It, revealed evidence of 6 highly credible events supported by significant *p*-values <0.001 and including 4 isolates (1 from Senegal (ArD85094) and 3 from Mauritania (ArD140166, ArD140187 and ArD140179)) (Table 2). The analysis with GARD identified 7 inferred breakpoints at positions 2849, 3215, 3912, 4489, 4842, 6860 and 9359 of WSLV genome (model average support values between 0.55 and 0.99). These recombination events included mainly the genomic regions encompassing the E, NS1 and NS5 proteins which have major roles in the replication of flaviviruses (Table 2).

**Table 2:** Recombination events identified on genomes of the newly characterized Wesselsbron virus isolates.

Event number	Recombinant sequence	Parent sequence	Genomic positions	region	Detection methods						
					R	G	B	M	C	S	T
**1**	ArD85094	ArD142143	2960-4057	NS1-NS2B	**+**	**+**	**+**	**+**	**+**	**-**	**+**
**2**	ArD140166	ArD140194	1864-2522	E-NS1	**-**	**+**	**-**	**+**	**+**	**+**	**+**
**3**	ArD140166	ArD140194	8232-8682	NS5	**+**	**+**	**+**	**+**	**+**	**-**	**+**
**4**	ArD140166	ArD65233	3355-3892	NS1-NS2A	**-**	**+**	**-**	**+**	**+**	**+**	**+**
**5**	ArD140187	ArD140194	1858-2773	E-NS1	**-**	**+**	**+**	**+**	**+**	**+**	**-**
**6**	ArD140179	ArD140194	1932-2288	E	**-**	**+**	**-**	**+**	**+**	**+**	**+**

**R:** RDP, **G:** Geneconv, **B:** BootScan, **M:** MaxChi, **C:** Chimaera, **S:** SiScan, and **T:** 3Seq.

### 3.4 Positive selection pressures

The structural and non-structural coding regions were analyzed separately for estimation of sites and branches under positive diversifying selection, applying four different methods to ensure consistency of these events along WSLV sequences. Using this approach, we found several sites under strong negative selection and most of them were in the NS5, NS3, NS1, E and NS4B proteins. However, significant evidence of positively selected sites or branches were detected decreasingly, in the NS3, NS4B, Capsid, E, and NS2A proteins (Table 3).

**Table 3:** Episodes of positive diversifying selection on Wesselsbron virus proteins.

Gene		Number of Sites detected by Method					Evidence of Positive Selection
		SLAC (*p* < 0.1)	FUBAR (Posterior Probability ≥ 0.9)	MEME (*p* < 0.1)	aBSREL (*p* < 0.05)	BUSTED (*p* < 0.05)	
Capsid	Sites under negative selection (dN/dS < 1)	0	5	0	0	0	YES
	Sites under positive selection (dN/dS > 1)	0	1	1	0	7	
prM/M	Sites under negative selection (dN/dS < 1)	5	16	0	0	0	NO
	Sites under positive selection (dN/dS > 1)	0	0	0	0	0	
E	Sites under negative selection (dN/dS < 1)	12	43	0	0	0	YES
	Sites under positive selection (dN/dS > 1)	0	2	1	0	1	
NS1	Sites under negative selection (dN/dS < 1)	23	71	0	0	0	NO
	Sites under positive selection (dN/dS > 1)	0	0	1	0	0	
NS2A	Sites under negative selection (dN/dS < 1)	9	26	0	0	0	YES
	Sites under positive selection (dN/dS > 1)	0	1	2	0	1	
NS2B	Sites under negative selection (dN/dS < 1)	4	9	0	0	0	NO
	Sites under positive selection (dN/dS > 1)	0	0	0	0	0	
NS3	Sites under negative selection (dN/dS < 1)	28	169	0	0	0	YES
	Sites under positive selection (dN/dS > 1)	0	2	7	2	11	
NS4A	Sites under negative selection (dN/dS < 1)	3	13	0	0	0	NO
	Sites under positive selection (dN/dS > 1)	0	0	0	0	3	
2K	Sites under negative selection (dN/dS < 1)	0	4	0	0	0	NO
	Sites under positive selection (dN/dS > 1)	0	0	0	0	0	
NS4B	Sites under negative selection (dN/dS < 1)	8	41	0	0	0	YES
	Sites under positive selection (dN/dS > 1)	0	0	2	1	7	
NS5	Sites under negative selection (dN/dS < 1)	44	262	0	0	0	NO
	Sites under positive selection (dN/dS > 1)	0	2	1	0	0	

Pervasive diversifying selection at posterior probability ≥ 0.9 with FUBAR model; Episodic diversifying selection at 0.1 significance level with SLAC and MEME models; Episodic diversifying selection at *p*-value *p* ≤ 0.05 with aBSREL and BUSTED models.

### 3.5 Evolutionary analyses

**Fig. 1:**
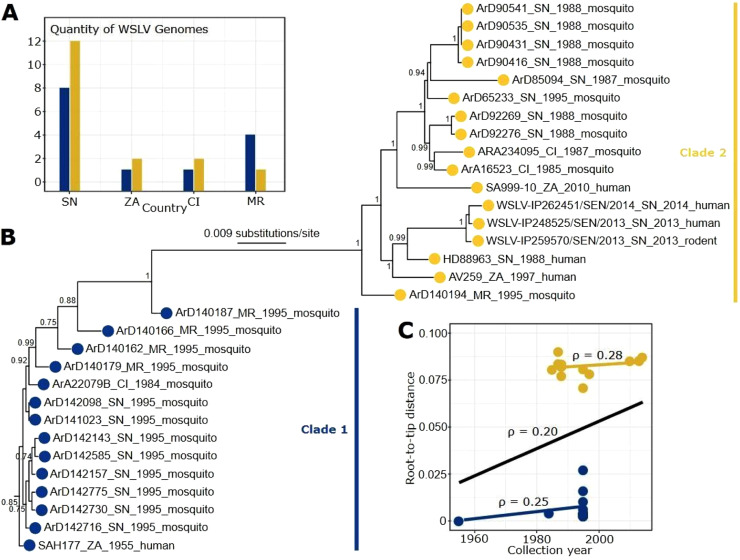
Maximum-Likelihood (ML) phylogenetic tree based on complete genome sequences of Wesselsbron virus from (A) four countries in Africa (SN, ZA, CI, MR). Tree branches are scaled by substitutions per site. Tree nodes show SH-like support values when > 0.7. Colored circles denote distinct clades. Tree tip labels include isolate/strain, country, and collection year. (C) A root-to-tip regression analysis using the data from (B). A black line shows the correlation coefficient (ρ) for all sequences. Colored lines estimate ρ for each clade.

Our datasets included the 25 newly characterized sequences from Senegal (SN), Mauritania (MR) and Côte d'Ivoire (CI) and previous sequences from Senegal and South Africa (ZA). Two distinct clades were estimated using a maximum-likelihood phylogenetic tree using all 31 coding complete regions. Both clades were identified in each country with distinct proportions (Fig. 1A). The tree was rooted to the 1955 primordial isolate from South Africa (ZA) (Fig. 1B). All the isolates identified since 1997 belonged to clade 2 (Fig. 1B). The tree exhibited that recombinant sequences grouped in both clade 1 (ArD140166, ArD140187 and ArD140179 from Mauritania) and clade 2 (ArD85094 from Senegal) (Fig. 1B) while each recombining with an isolate belonging of the other clade (Table 2), suggesting the occurrence of inter-clade recombination in WSLV. The temporal signal among WSLV strains was estimated using a root-to-tip regression analysis. The correlation coefficient (ρ) for all sequences was 0.20, suggesting a weak correlation between genomic distance and time of collection. The correlation improved slightly when the clades were analysed individually (Fig. 1C).

### 3.6 Viral fitness

#### 3.6.1 Survival rates

**Fig. 2:**
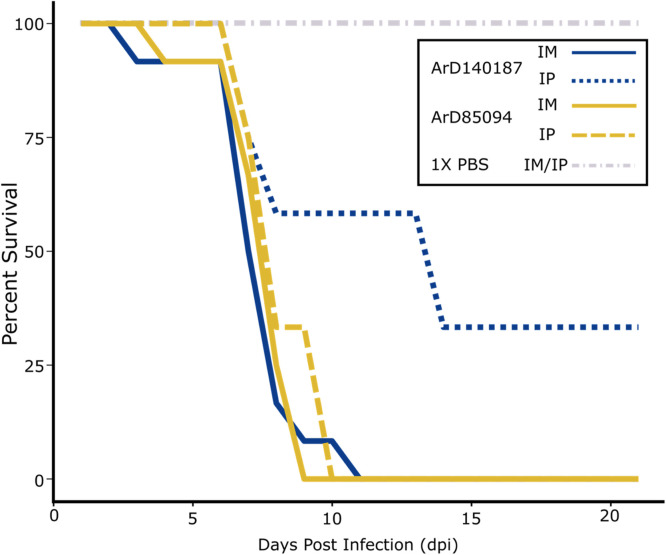
Survival curves of 3-week-old (wko) mice following intramuscular (IM) and intraperitoneal (IP) infection with 100,000 PFU of clade 1 (ArD85094) and clade 2 (ArD140187). Mice were monitored daily for 21 days. All survival curves were significantly different to the control groups (grey dashed line) (Wilcoxon rank sum test, *p*-values < 0.05).

All animals in the 3-wko Swiss adult mice inoculated groups and the controls groups survived over the 21-days study-period. Mortality was observed in the different Swiss suckling mice inoculated groups from dpi 3 to dpi 14. The recombinant isolate ArD85094 (clade 1) was pathogenic for mice inoculated through i.m and i.p routes and caused an observed mortality of 100% for both inoculation routes between dpi 4 and dpi 9 and dpi 7 and dpi 10, respectively. In addition, the isolate ArD140187 (clade 2) caused 100% of mortality in groups inoculated by i.m route from dpi 3 to dpi 11 and showed 34% of survival in groups inoculated by i.p route between dpi 7 and dpi 14. The difference observed in survival rates between the group inoculated by i.p with the isolate ArD85094 (clade 1) and that inoculated by the same route with the ArD140187 (clade 2), was statistically significant (Mantel-Cox test *p*-value <0.0001 and Gehan-Breslow-Wilcoxon test *p*-value = 0.001). Differences observed in survival rates between inoculated and control groups were statistically significant (Mantel-Cox test *p*-value <0.0001 and Gehan-Breslow-Wilcoxon test *p*-value = 0.0006) (Fig. 2).

#### 3.6.2 Clinical signs in suckling mice

No body weight change was observed in challenged mice. Mice from groups inoculated with the recombinant isolate ArD85094 (clade 1) developed symptoms of encephalitis that included hunched posture, limb paralysis, ataxia, weakness, tremor, reduced mobility and incoordination of movements while those inoculated with the recombinant isolate ArD140187 (clade 2) exhibited only limb paralysis and tremor. These symptoms were observed from dpi 3 to dpi 9 and were not inoculation route-dependent.

#### 3.6.3 Viral neurotropism in Swiss suckling mice

All isolates were 100% lethal in Swiss suckling mice with a mortality from dpi 4 to dpi 21.The viral RNA was detected in brain tissues of mice inoculated with the isolate ArD85094 (clade 1) by i.m route from dpi 4 to dpi 9 with an increasing viral load (ranging from 1.27×10^3^ to 7.60×10^7^ copies/uL) while for those inoculated with the same isolate through the i.p route, it was detected from dpi 7 to dpi 9 (ranging from 6.97×10^5^ to 2.41×10^8^ copies/uL). For mice inoculated with the isolate ArD140187 (clade 2) through the i.m route, the viral RNA was detected in brain tissues from dpi 4 to dpi 8 with an increasing viral load (ranging from 1.27×10^3^ to 7.07×10^8^ copies/uL) while the i.p route showed a presence of viral RNA in the brain from dpi 8 to dpi 21 with a decreasing viral load over the monitoring time (ranging from 1.60×10^3^ to 6.60×10^8^ copies/uL). In addition, a decreasing viral load was recorded for mice inoculated with the isolate ArD140187 (clade 2) through i.m route which collected at dpi 14 and 21 (5 log of reduction from dpi 8 to dpi 21) (Table 4).

**Table 4:** Assessment of Wesselsbron virus neurotropism in Swiss suckling mice over dpi 21.

Isolate	ArD85094 (clade 1)		ArD140187 (clade 2)	
Inoculation route	i.m	i.p	i.m	i.p
**Day post infection**	**RT-qPCR results (Copy numbers)**			
dpi 4	Positive (1.27×10^3^)	-	Positive (1.27×10^3^)	-
dpi 7	Positive (4.11×10^7^)	Positive (6.97×10^5^)	Positive (4.09×10^6^)	Positive (4.12×10^8^)
dpi 8	Positive (4.79×10^7^)	Positive (2.41×10^8^)	Positive (7.07×10^8^)	Positive (6.06×10^8^)
dpi 9	Positive (7.60×10^7^)	Positive (2.05×10^7^)	-	-
dpi 14	-	-	-	Positive (1.38×10^4^)
dpi 21	-	-	-	Positive (1.60×10^3^)

i.m: intra-muscular i.p: intra-peritoneal dpi: day post-infection

#### 3.6.4 Viral tropism in Swiss adult mice

The virus wasn't detected in any organs tissues from euthanized of mice inoculated with the isolate ArD140187 (clade 2) through i.m route over dpi 21 while it was detected at dpi 7 in the blood from mice inoculated with the same isolate through i.p route. Interestingly, the virus dissemination was observed only for the isolate ArD85094 (clade 1) inoculated through the i.m route. The viral RNA was detected in blood, brain tissues, lungs and kidneys at dpi 7 and dpi 14, with a viral load decrease (of 5 Log) in brain tissues at dpi 14 and a viral load increase (of 6 Log) in the lungs. In addition, RNA was also detected in the liver tissues only at dpi 7. The highest viral load was observed in the blood, the brain tissues and the lungs with a median copy number of 6.40×10^3^, 3.20×10^3^ and 1.37×10^3^ copies/uL, respectively. However, for mice inoculated with the isolate ArD140187 (clade 1) through the i.p route, the viral RNA was only detected in the blood at dpi 1 and the lungs at dpi 7 (Table 5).

**Table 5:** Viral loads in organs tissues of adult mice at dpi 1, 7, 14 and 21.

Inoculation route	Tissue	Wesselsbron viral load in organ tissues per isolate					
		**ArD85094 (clade 1)**			**ArD140187 (clade 2)**		
		detection period	median copy number	SD (log_10_)	detection period	median copy number	SD (Log_10_)
i.m	Blood	dpi 7 to dpi 14	6.40×10^3^	-	-		
	Brain	dpi 7 to dpi 14	3.20×10^3^	5^Δ^	-		
	Lungs	dpi 7 to dpi 14	1.37×10^3^	6*	-		
	Liver	dpi 7	40	-	-		
	Kidneys	dpi 7 to dpi 14	7.44×10^2^	-	-		
i.p	Blood	dpi 1	2.01×10^2^	-	dpi 14	2.74×10^2^	-
	Brain	-			-		
	Lungs	dpi 7	80	-	-		
	Liver	-			-		
	Kidneys	-			-		

SD: standard deviation i.m: intra-muscular i.p: intra-peritoneal dpi: day post-infection ^Δ^: decrease of viral load *: increase of viral load

## 4 Discussion

Wesselsbron disease is a neglected, mosquito-borne infection of public health and economic importance reported in Africa (Diagne et al., 2017; Weyer et al., 2013), for which the real burden remains sparsely determined (Diagne et al., 2017; Weyer et al., 2013; Swanepoel, 1989; Gould et al., 2003). Only rare data are currently available and the identification of WSLV infection is difficult in Africa (McIntosh, 1980) where limited resources, infrastructure and diagnostic capacities exist. In this study, we investigated not only the molecular epidemiology of WSLV in Africa using newly characterized whole genome sequences from three West African countries, but also its pathogenicity and tropism using in vivo experiments in mice. These provide new insights in the viral genetic diversity that could be useful for prevention, preparedness and future outbreak response.

Low amino acid distances observed between WSLV sequences analysed in our study (<2%) in comparison with previously available African sequences confirmed a low genetic diversity of WSLV as previously described (Diagne et al., 2017).

Although their role in altering functional properties of the flavivirus E protein which increase neurovirulence is not well understood, mutations in the E protein of WSLV are presumed to be important (Davis et al., 2022). Mutations in the E protein were previously reported from a rare neurovirulent revertant of the YF17D vaccine strain (Jennings et al., 1994); assuming that receptor binding and subsequent events associated with virus entry, including low-pH-induced conformational changes and fusion with intracellular membranes, are principally involved. Nevertheless, mutations at numerous positions of the domains I, II and III of the E protein reduced the virulence properties for encephalitic flaviviruses which have been characterized in mice (McMinn, 1997; Guirakhoo et al., 2004). However, the impact of polymorphisms in virulence determinants in the non-structural regions of WSLV needs to be better investigated as they are likely to affect enzymatic activities associated with viral transcription, translation of the viral polyprotein, or virion assembly or to RNA structures involved in cis-acting regulatory events and/or interactions with host proteins (Muylaert et al., 1996). The RDG motif of the EDIII was previously identified as a determinant of vector specificity for MVBFs (Hung et al., 2004), non-conserved in members of the Dengue virus serocomplex primarily transmitted by *Aedes* mosquitoes (Huang et al., 2014), such as WSLV. Playing a role in virus entry and viral infectivity, its polymorphism led to higher *in vitro* infection rates for the Asibi YFV strain (Huang et al., 2014). Previously associated with the teratogenic effect observed in ZIKV infection (Shan et al., 2020), the modified E-V459M substitution identified for the first time in the WSLV envelope protein (E-V459I) could be involved in maternal-to-fetal transmission and abortion. However, the impact of these two motifs of virulence in the WSLV pathogenicity needs to be further investigated. These findings provide insights into the genetic profile of Wesselsbron virus and a potential target for broad-spectrum antivirals and vaccine design.

Although recombination was previously documented in other members of the mosquito-borne flavivirus group (Kuno et al., 1998; Simon-Loriere and Holmes, 2011), our study is noteworthy by identifying, for the first time, six recombination events in major proteins of WSLV such as the E, NS1 and NS5 protein. Our data provide evidence that recombination events occur in WSLV. The identification of natural recombination events between WSLV provides new insights in the epidemiology of this neglected mosquito-borne zoonotic disease, widely distributed in Africa (Karabatsos, 1985). Although Recombination is rarely observed in flavivirus genomes (Taucher et al., 2010), the occurrence of inter-clade recombination in WSLV could be associated with co-circulation of both clades in each of these countries. However, recombination occurs only when at least two viral genomes co-infect the same host cell simultaneously and exchange genetic segments by an unknown mechanism in the cell cytoplasm, resulting in the generation of recombinant virus (Pérez-Losada et al., 2015). Despite the very low frequency of intergenomic recombination in the structural region of flaviviruses (Taucher et al., 2010), our data also identified for the first time, recombination in the E protein of WSLV. The E protein is highly important because it encodes the most important antigen with regards to virus biology and humoral immunity. It is involved in cell receptor recognition, attachment, cell fusion, tropism, and virulence (Roby et al., 2015). Recombination has been previously described on the E protein of ZIKV (Faye et al., 2014). The entry of flaviviruses into their target cells is mediated by the interaction of the E glycoprotein with cell surface receptors. Through that process, glycosaminoglycans (GAGs) act mainly as attachment factors that concentrate flavivirus particles at the target cell surface before their interaction with primary receptors (Perera-Lecoin et al., 2014). However, more investigations for identification of the cellular receptors that mediate flavivirus entry and infection are warranted. Though the precise molecular mechanisms of the template switches are unknown, occurrence of recombination breakpoints in the E protein of WSLV could have significant impact on virus phenotype (Twiddy and Holmes, 2003). Associated with the other non-structural proteins, the NS1 protein plays an important function in viral replication and assembly and viral escape to host innate immune response. It is the most conserved non-structural protein of flaviviruses (Rastogi et al., 2016). The NS5 protein is the largest viral protein that serves as the RNA-dependent RNA polymerase (RdRp) and performs multiple functions essential for viral replication (Egloff et al., 2007). As recently described for Dengue virus 2 and 4, recombination in the NS5 protein may contribute to the emergence of a new distinct phylogenetic clade (Wardhani et al., 2023) or affect replication capacity (Hwang et al., 2020). This interclade recombination could result in the emergence of novel strains with altered pathogenic potential and antigenicity. Our data provide insightful information on the virus evolution, which could be useful for prevention, preparedness and future outbreak response. The presence of positive selective sites in the NS3, NS4B and Capsid protein suggest that they could represent preferential selection targets during WSLV evolution (Carney et al., 2012). The NS3 and NS4B are involved in the induction of strong innate immunity suppression mechanisms (Gebhard et al., 2016), viral adaptation (Maringer and Fernandez-Sesma, 2014), pathogenesis (Miller et al., 2006), neurovirulence (Zmurko et al., 2015) and host preferences (Han et al., 2014). Although, positive selection episodes have been previously reported for the DENV-3 Capsid (Chwan-Chuen et al., 2008), their impact on WSLV's pathogenicity needs to be further investigated as the Capsid protein of flaviviruses contain all the cis-acting RNA elements required for viral RNA replication (Baker et al., 2020).

The phylogenetic analysis suggests that WSLV has undergone a distinct evolutionary event that resulted in two distinct clades, with clade 1 preceding the emergence of clade 2 and all strains isolated since 1997 belonging to clade 2. However, the slightly reduced root-to-tip regression value of 0.2 when combining both clades, could be associated with the potential biases due recombination during phylogenetic inference (Schierup and Hein, 2000). Our study is noteworthy by providing insightful data that will contribute to better understanding the recent molecular epidemiology of WSLV in Africa for preparedness and response to probable future outbreaks. More frequently isolated from *Aedes* mosquitoes which are also vectors for flaviviruses of public health concern such as Zika, Yellow fever and Dengue (Heinz and Stiasny, 2017; Huang et al., 2014; Shan et al., 2020), increase in the prevalence of WSLV in mosquito populations could be associated to its spillover in vertebrates (Diagne et al., 2017; Weyer et al., 2013; Durand et al., 2001; McIntosh, 1980). In addition, this shift in the evolutionary dynamic of WSLV could have been enhanced by the introduction of non-African *A. aegypti* mosquitoes since early 2000s (Rose et al., 2020) and the progressive invasion of *A. albopictus* across Africa since 1999 (Longbottom et al., 2023), probably related to urbanisation and climate changes (Baker et al., 2020; Mordecai et al., 2020). This change in the dispersal dynamics of *Aedes* vectors could have largely contributed to the increasing number of arbovirus infections transmitted by *Aedes* vectors reported in West Africa since 2007 (Buchwald et al., 2020) and WSLV spillover in humans ten years ago in Senegal (Diagne et al., 2017) and WSLV re-emergence in areas where it was missed over five decades (Oymans et al., 2020). However, since the distribution of *Aedes* subgenera tends to follow a geographic pattern in Africa with both *Ae. (Aedimorphus)* and *Ae. (Stegomyia)* predominating in West Africa (Swanepoel, 1989; Kraemer et al., 2015), it is important to promote more experimental studies focusing on the identification of the Ae. vectors involved in WSLV maintenance and transmission to humans and gather useful information to forecast risk, facilitate preparedness and response or control of future outbreaks. Given the relatively few complete WSLV genome sequences previously available in GenBank prior to our study (n= 6) (https://www.ncbi.nlm.nih.gov/nuccore/?term=%22wesselsbron%22+%22complete+genome%22; accessed on 02 February 2024), this additional genetic information will help guide assay update nor countermeasure development efforts for this important but neglected virus.

Although the most recent isolations and spillover in humans involved the clade 2 (Diagne et al., 2017; Weyer et al., 2013; Kayiwa et al., 2022), the clade 1 appears to be more pathogenic in suckling mice, exhibiting more symptoms of encephalitis. These data could suggest a probable silent circulation of the clade 2 WSLV during the last decade with less severe cases. However, these data could be further confirmed using more isolates from both clades. Nevertheless, active entomological including genomic sequencing and serology-based veterinary and clinical surveillance studies could be ruled out in regions where the *Aedes* vectors have been previously identified (Weinbren, 1959; Kokernot et al., 1960; Kokernot et al., 1958; Diallo et al., 2005; Huhtamo et al., 2009) to identify potential reservoirs and estimate the real public and veterinary health impact of this disease in Africa. However, both clades are neurotropic in suckling mice and produce high viral loads in brain tissues when inoculated through i.m route, which is the most likely horizontal transmission mode for arboviruses including WSLV (Lambrechts and Scott, 2009). Although no mortality was observed in adult mice, the clade 1 inoculated through the i.m route, disseminated and exhibited tropism for vital organs such as the brain, lungs and kidneys. These data suggest that WSLV is then able to cross the blood-brain barrier (BBB) in adult mice such as West Nile (WNV) and Japanese encephalitis virus (JEV) (Pardigon, 2017). However, its capability of neuroinvasion and neurovirulence in adult mice need to be assessed through further experimental studies in order to highlight possible mechanisms involved in WSLV neuropathogenesis. In addition, possible similar pathophysiological features to Zika virus, which very rarely reaches the brain of adults, but can infect neural progenitors in the developing central nervous system of fetuses, could be also investigated by analysing age-specific differences in the response of brain capillary endothelial cells (BCECs) to WSLV infection for better understanding of devastating congenital complications including abortion (Pardigon, 2017; Basu et al., 2021). The absence of virus dissemination in adult mice inoculated through the i.p route could be correlated with the capacity of peritoneal macrophages from adult mice to rapidly inactivate the WSLV infectivity as previously described (Olson et al., 1975).

Infecting humans, Wesselsbron differential diagnosis should be considered in a context of investigation for other arbovirus infections such as those including Rift valley fever (Swanepoel, 1989; Gould et al., 2003). Beyond a health concern, outbreak of WSLV could also, more severely affect incomes of impoverished individuals and families of semi-nomadic pastoralists by directly and indirectly causing long-lasting and devastating effects on the quality of life as described for RVFV (Friel et al., 2004). Overall, we identified, for the first time, inter-clade recombination events on the genome of WSLV. Although no geographical correlation was found on the phylogenetic data, the clade 1 was more pathogenic and neurotropic in suckling mice and the intramuscular route was found to be the best transmission mode. Our findings provide new insights in the pathogenicity and tropism of WSLV which could be useful for prevention, preparedness and future outbreak response. However, more serological and experimental studies should be promoted to assess the potential for WSLV to sustain epidemic transmission among humans as other neurotropic flaviviruses such as WNV, ZIKV and JEV (Pierson and Diamond, 2020). Closely related to *Aedes sp.-*transmitted human pathogenic flaviviruses such as YFV and ZIKV (Heinz and Stiasny, 2017; Huang et al., 2014, Shan et al., 2020), the potential risk of WSLV spread outside of Africa warrants more attention, considering the global distribution of the two most prolific *Aedes* mosquito vectors (Kraemer et al., 2015; Laporta et al., 2023).

## Funding

This research received no specific grant from any funding agency in the public, commercial, or not-for-profit sectors and was only supported by the Institut Pasteur de Dakar internal funds for research.

## Author statement

The authors declare that they have no competing financial interests. The supporting sponsors had no role in the design of the study; in the collection, analyses, or interpretation of data; in the writing of the manuscript, and in the decision to publish the results.

## CRediT authorship contribution statement

**Martin Faye:** Writing – review & editing, Writing – original draft, Visualization, Validation, Supervision, Software, Resources, Project administration, Methodology, Investigation, Formal analysis, Data curation, Conceptualization. **Nicholas Di Paola:** Writing – original draft, Visualization, Validation, Software, Formal analysis. **Moussa Dia:** Writing – review & editing, Methodology, Investigation. **Amadou Alpha Sall:** Writing – review & editing, Supervision, Funding acquisition, Conceptualization. **Ousmane Faye:** Writing – review & editing, Supervision, Funding acquisition, Conceptualization.

## Declaration of competing interest

The authors declare that they have no known competing financial interests or personal relationships that could have appeared to influence the work reported in this paper.

## Data Availability

All data are present in this manuscript and the newly characterized sequences are available on Genbank (www.ncbi.nlm.nih.gov/genbank/) under accession numbers PP445078- PP445102.

## Appendix Supplementary materials

Supplementary Figure S1: Polymorphism on the envelope protein V473M substitution (E-V473M; motif LL**M**WLGLN between aa positions 471-478) identified at amino acid position 459 in the E protein of the newly characterized Wesselsbron virus sequences and replaced by the Methionine was replaced by an isoleucine (E-M459I; motif LL**I**WLGLN between aa positions 457-464). It has been previously identified in ZIKV and associated with an increased neurovirulence, maternal-to-fetal transmission, and viremia to facilitate urban transmission (Martin et al., 2015). Image, image 1

Image, application 2
