# Supplementary figures and images for: Molecular epidemiology and pathogenicity of Wesselsbron virus circulating in Africa
Source: Virus Res. 2024 Nov 17;350:199499. doi: 10.1016/j.virusres.2024.199499 (PMC11615586; doi:10.1016/j.virusres.2024.199499)

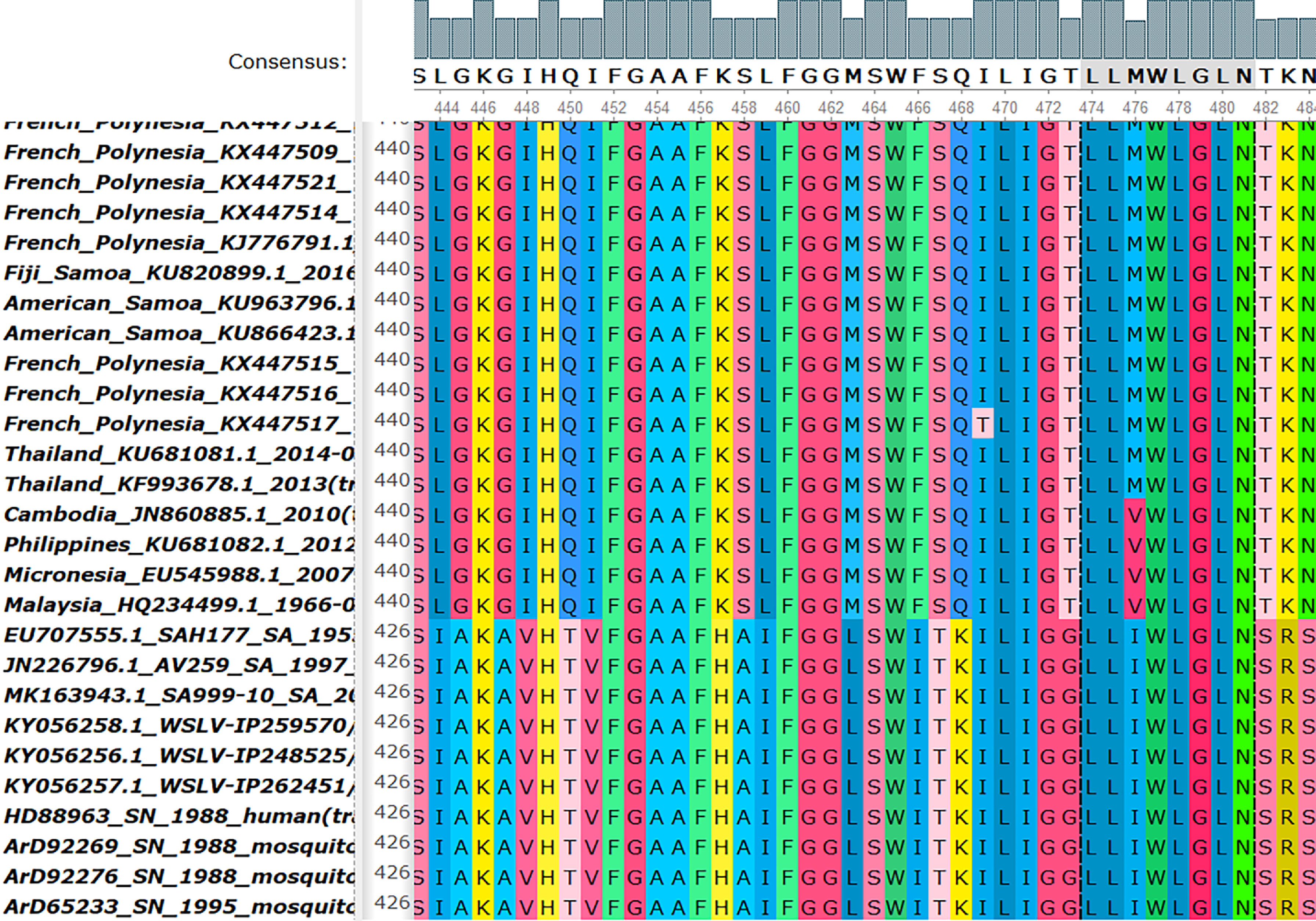

Supplement: Supplementary file 1 — Supplementary Figure S1: Polymorphism on the envelope protein V473M substitution (E-V473M; motif LLMWLGLN between aa positions 471-478) identified at amino acid position 459 in the E protein of the newly characterized Wesselsbron virus sequences and replaced by the Methionine was replaced by an isoleucine (E-M459I; motif LLIWLGLN between aa positions 457-464). It has been previously identified in ZIKV and associated with an increased neurovirulence, maternal-to-fetal transmission, and viremia to facilitate urban transmission (Martin et al., 2015). [file mmc1.jpg]
