# Supplementary material for: Molecular epidemiology and pathogenicity of Wesselsbron virus circulating in Africa
Source: Virus Res. 2024 Nov 17;350:199499. doi: 10.1016/j.virusres.2024.199499 (PMC11615586; doi:10.1016/j.virusres.2024.199499)
Supplement: Supplementary file 2 [file mmc2.docx]

**Supplementary materials**

**Table S1.** Information on Wesselsbron virus isolates characterized in this study.

| **Isolate** | **Country** | **Year of isolation** | **Specie** | **Genbank accession numbers** |
| --- | --- | --- | --- | --- |
| ArD141023 | Senegal | 1995 | *Aedes dalieli* | PP445078 |
| HD88963 | Senegal | 1988 | *Homo sapiens* | PP445079 |
| ArD92269 | Senegal | 1988 | *Cellia domicola* | PP445080 |
| ArD92276 | Senegal | 1988 | *Cellia pharoensis* | PP445081 |
| ArA16523 | Côte d’Ivoire | 1985 | *Culex perfuscus* | PP445082 |
| ArD142585 | Senegal | 1995 | *Aedes dalieli* | PP445083 |
| ArD142157 | Senegal | 1995 | *Aedes dalieli* | PP445084 |
| ArD85094 | Senegal | 1987 | *Aedes dalieli* | PP445085 |
| ArD142143 | Senegal | 1995 | *Aedes dalieli* | PP445086 |
| ArA22079B | Côte d’Ivoire | 1984 | *Stegomyia africanus* | PP445087 |
| ARA234095 | Côte d’Ivoire | 1987 | *Aedes abnormalis* | PP445088 |
| ArD65233 | Senegal | 1985 | *Aedes dalieli* | PP445089 |
| ArD90416 | Senegal | 1988 | *Stegomyia luteocephalus* | PP445090 |
| ArD90431 | Senegal | 1988 | *Aedes vittatus* | PP445091 |
| ArD90541 | Senegal | 1988 | *Aedes minutus* | PP445092 |
| ArD90535 | Senegal | 1988 | *Aedes minutus* | PP445093 |
| ArD140194 | Mauritania | 1995 | *Aedes vexans* | PP445094 |
| ArD142098 | Senegal | 1995 | *Aedes dalieli* | PP445095 |
| ArD140166 | Mauritania | 1995 | *Aedes vexans* | PP445096 |
| ArD140179 | Mauritania | 1995 | *Aedes vexans* | PP445097 |
| ArD140162 | Mauritania | 1995 | *Aedes vexans* | PP445098 |
| ArD140187 | Mauritania | 1995 | *Aedes vexans* | PP445099 |
| ArD142716 | Senegal | 1995 | *Aedes dalieli* | PP445100 |
| ArD142730 | Senegal | 1995 | *Aedes dalieli* | PP445101 |
| ArD142775 | Senegal | 1995 | *Aedes dalieli* | PP445102 |

**Table S2.** Location of highly conserved flavivirus amino acid motifs across the Envelope, NS1, NS3 and NS5 proteins of Wesselsbron virus.

| **Gene** | **Amino Acid Motifs Previously Described in MBFVs ^#^** | **Cons * on Used NKVFs/ISFs Genome** | **Positions on WSLV Genome** | **Cons * on WSLV** | **Except on These WSLV Isolates** | **Replaced by This Consensus Sequence** |
| --- | --- | --- | --- | --- | --- | --- |
| **E** | DRGWGNGC | **YES** | 378–385 | **YES** | 14 isolates from SN, MR and CI | DRGWGN**S**C |
|  | GLFGKGS | only on NKVFs | 386–392 | **YES** |  |  |
|  | GHLKCRV | **NO**  RBV  (GH**VD**CRV)  ModV (GHV**S**C**K**V) | 556-562 | **NO** | All isolates | GH**VT**C**KA** |
|  | PFGDSYIV | NO | 646–655 | **YES** | All isolates | PFG**E**SYI**I** |
| **NS1** | DTAWDFGS | NO | 692–699 | **NO** | All isolates | **EHS**WDFGS |
|  | GCWYGMEI | only on NKVFs | 1100–1105 | **YES** | All isolates | GCWY**S**ME**V** |
|  | YGMEIRP | YES | 1102–1108 | **YES** | All isolates | Y**S**ME**V**RP |
| **NS3** | GTSGSPI | YES | 1614–1620 | **YES** | 5 isolates from SN and ZA | G**S**SGSPI |
|  | GLYGNG | only on NKVFs and CxFV | 1629–1634 | **YES** |  |  |
|  | LAPTRVV | YES | 1705-1711 | **YES** |  |  |
|  | DVMCHATF | Only on NKVFs | 1742–1749 | **YES** | All isolates | D**A**MCHAT**L** |
|  |  |  |  |  |  |  |
|  | MDEAHF | YES | 1767–1772 | **YES** |  |  |
|  | SIAARGY | YES | 1777–1783 | **YES** | 29 isolates from SN, ZA, MR and CI | SIAARG**W** |
|  |  |  |  |  | ArD85094_SN_1987_mosquito and ArA22079B_CI_1984_mosquito | **IM**A**DI**G**W** |
|  | MTATPPG | YES | 1798–1804 | **YES** | All isolates | M**S**ATPPG |
|  | ISEMGAN | YES | 1894–1900 | **YES** | All isolates | I**A**EMGAN |
|  | SAAQRRGR | YES | 1936–1943 | **YES** |  |  |
| **NS5** | DLGCGRG | YES | 2578–2584 | **YES** |  |  |
|  | SRNSTHEMY | YES | 2711–2719 | **YES** | ArD142157_SN_1995_mosquito | SRNSTHE**R**Y |
|  | NMMGKREKK | YES | 2953–2961 | **YES** |  |  |
|  | ADDTAGWDT | YES | 3032–3040 | **YES** |  |  |
|  | WMTTEDML | YES | 3306–3313 | **YES** |  |  |

Cons *: conservation of motif; YES: conserved motif; NO: non-conserved motif; WSLV: Wesselsbron virus; ISFs: insect-specific flaviviruses; Culex flavivirus (CxFV) and Aedes Flavivirus (AeFV); NKVFs: no-known vector flaviviruses ; Modoc virus (ModV) and Rio Bravo virus (RBV); **^#^**: Conserved amino acid motifs as described in mosquito-borne flaviviruses (MBVFs) by Kuno, G.; Chang, G.J. 2007 (32); Positions with amino acid different on BAGV polyprotein are highlighted in black for conservative mutations and in red for non-conservative mutations; Isolates name is labeled in the following format: Identification number, Origin, country code (SN: Senegal, CI: Côte d’Ivoire, MR: Mauritania, ZA: South Africa), and year of isolation; Except for the Indian Isolate (name, country code, year of isolation and host).
